# Targeting Heme Oxygenase 2 (HO2) with TiNIR, a Theragnostic Approach for Managing Metastatic Non-Small Cell Lung Cancer

**DOI:** 10.34133/bmr.0026

**Published:** 2024-04-25

**Authors:** Seul-Ki Mun, Hyun Bo Sim, Jae-Hyuk Lee, Hyeongyeong Kim, Dae-Han Park, Yong-An Lee, Ji Yeon Han, Yu-Jeong Choi, Jun Sang Son, Jeongwon Park, Tae-Hwan Lim, Sung-Tae Yee, Young-Tae Chang, Seongsoo Lee, Dong-Jo Chang, Jong-Jin Kim

**Affiliations:** ^1^ Department of Biomedical Science, Sunchon National University, Suncheon 57922, Republic of Korea.; ^2^ College of Pharmacy and Research Institute of Life and Pharmaceutical Sciences, Sunchon National University, Suncheon 57922, Republic of Korea.; ^3^ Gwangju Center, Korea Basic Science Institute (KBSI), Gwangju 61751, Republic of Korea.; ^4^ Genome Institute of Singapore (GIS), Agency for Science, Technology and Research (A*STAR), 60 Biopolis Street, Genome, Singapore 138672, Republic of Singapore.; ^5^ School of Interdisciplinary Bioscience and Bioengineering, Pohang University of Science and Technology (POSTECH), Pohang 37673, Republic of Korea.; ^6^ Department of Chemistry, Pohang University of Science and Technology (POSTECH), Pohang 37673, Republic of Korea.; ^7^ Department of Systems Biotechnology, Chung-Ang University, Anseong 17546, Republic of Korea.; ^8^ Department of Bio-Analysis Science, University of Science & Technology, Daejeon 34113, Republic of Korea.

## Abstract

Despite notable advancements in cancer therapeutics, metastasis remains a primary obstacle impeding a successful prognosis. Our prior study has identified heme oxygenase 2 (HO2) as a promising therapeutic biomarker for the aggressive subsets within tumor. This study aims to systematically evaluate HO2 as a therapeutic target of cancer, with a specific emphasis on its efficacy in addressing cancer metastasis. Through targeted inhibition of HO2 by TiNIR (tumor-initiating cell probe with near infrared), we observed a marked increase in reactive oxygen species. This, in turn, orchestrated the modulation of AKT and cJUN activation, culminating in a substantial attenuation of both proliferation and migration within a metastatic cancer cell model. Furthermore, in a mouse model, clear inhibition of cancer metastasis was unequivocally demonstrated with an HO2 inhibitor administration. These findings underscore the therapeutic promise of targeting HO2 as a strategic intervention to impede cancer metastasis, enhancing the effectiveness of cancer treatments.

## Introduction

Cancer represents a substantial global health threat, contributing substantially to mortality rates [1]. Despite extensive efforts employing diverse treatment modalities, such as apoptosis induction, inhibition of metastasis and proliferation, and immune response regulation [2,3], patients grapple with challenges in sustaining life amidst ongoing disease progression. While surgical intervention is a potentially curative option, often results in tumor recurrence and metastasis after resection [4–6]. Moreover, despite chemotherapy, up to 50% of patients experience recurrence and metastasis within 1 year of surgery owing to the emergence of drug resistance [7]. Thus, the identification of an efficient therapeutic biomarker and corresponding drug capable of preventing cancer progression is imperative for devising a successful strategy for cancer therapy.

In our previous study, we developed tumor-initiating cells (TICs)-specific probe, Tumor Initiating cell probe with Near Infra-Red (TiNIR), through unbiased cell-based screening of fluorescent chemical compound libraries in well-established TIC models. TICs, also known as cancer stem cells, a subset believed to drive cancer initiation and progression, were selectively targeted by TiNIR. Rigorous validations underscored its exceptional selectivity toward TICs, which was attributed to its binding with heme oxygenase 2 (HO2). HO2, a critical enzyme in cellular homeostasis, plays a pivotal role in converting heme to biliverdin, subsequently metabolized to bilirubin [8–10]. HO2 also generates CO and Fe^2+^, which can regulate various physiological events, participating in the biliverdin and bilirubin redox cycles for the removal of reactive oxygen species (ROS). Notably, HO2 is highly expressed in TICs, suggesting its reliability as a TIC marker and a potential therapeutic target. Inhibition of HO2 through TiNIR treatment triggers ROS accumulation, inducing apoptosis of TICs [10]. Despite these insights, the functional role of HO2 and the therapeutical impact of its inhibition in cancer progression remain unclear, warranting further exploration.

Strategic therapeutic approaches to control the expansion and metastasis of cancer involve targeting the cell cycle and migration [3]. AKT, a well-known factor in oncogene signaling, crucially regulates the characteristics of cancer cells, including cell survival and metastasis metabolism [11,12]. Consequently, the inhibition of AKT phosphorylation (p-AKT) is a significant therapeutic approach for cancer treatment. AKT influences proliferation, metastasis, and angiogenesis in the progression of the cancer cycle by regulating downstream signals, such as P21, cyclin-dependent kinase 1 (CDK1), and cyclin B. Activation of AKT ultimately leads to the phosphorylation of CDK1 and cyclin B, promoting the progression of the cell cycle [12,13]. Additionally, tuberous sclerosis 1 (TSC1) inhibits cell growth via AKT-mediated phosphorylation [11–13]. JUN signaling also regulates the G2/M phase of the cell cycle by mediating cyclin B1 and CDK2 [14,15]. Furthermore, AKT also plays a regulatory role in cell migration by controlling tubulin βIII (*TUBB3*), which facilitates cell–cell or cell–extracellular matrix interactions necessary for cellular movement [16,17]. Therefore, AKT and Jun signaling pathways are critical targets for managing metastatic cancer.

In this study, we investigated the potential utility of HO2 as a therapeutic biomarker for tracking and treating metastatic cancer [18]. Employing robust lung metastatic cancer models, we evaluated the therapeutic impact of targeting HO2 in cancer metastasis through in vitro and in vivo models. Our exploration aimed to unravel the molecular mechanism behind the therapeutic effect of targeting HO2 revealed that HO2 inhibition regulates the AKT and cJUN signaling pathways, impacting cell cycle progression and migration. Furthermore, inhibition of HO2 via repeated TiNIR treatments successfully prevented the formation of metastatic tumors in the lungs of metastatic cancer mouse models. These findings demonstrate that HO2 functions as a therapeutic biomarker, suggesting that the inhibition of HO2 is a promising approach in treating metastatic cancer.

## Materials and Methods

### Cell cultures

Human lung carcinoma cells (A549, KCLB 10185), human gastric carcinoma cells (AGS, KCLB 21739), human colon carcinoma cells (HCT116, KCLB 10247), human hepatoblastoma cells (HepG2, KCLB 88065), and human breast adenocarcinoma cells (MCF-7, KCLB 3002) were acquired from the Korean Cell Line Banks (Seoul, Republic of Korea). Human melanoma adenocarcinoma cells (MDA-MB-435s) were obtained from JW-pharma (Seoul, Republic of Korea) The cells were cultured in the presence of 10% fetal bovine serum (FBS, Hyclone, CA, USA), 2-mercaptoethanol (Thermo, MA, USA), Hepes (Thermo, MA, USA), sodium pyruvate, antibiotic–antimycotic in Dulbecco's Modified Eagle Medium (Hyclone, CA, USA) or RPMI 1640 medium (Hyclone, CA, USA). Cultures were maintained at 37 °C and 5% CO_2_.

### Quantitative real-time PCR

The total RNA from cell lines was purified using an RNeasy Mini kit (Qiagen, Valencia, CA, USA). RNA was eluted in ribonuclease (RNase)-free water, quantified using a NanoDrop 2000 (Thermo, DE, USA), and diluted 300 ng/3.5 μl in RNase-free water. Real-time polymerase chain reaction (PCR) using RNA-direct SYBR® Green Realtime PCR Master Mix kit was performed according to the manufacturer instructions with modification.

The quantitative PCR (qPCR) primers are as follows:

Glyceraldehyde 3-phosphate dehydrogenase (GAPDH) Forward: GTCTCCTCTGACTTCAACAGCG,

GAPDH Reverse: ACCACCCTGTTGCTGTAGCCAA,

HO2 Forward: GGACAGGCGACAGCGAC,

HO2 Reverse: CCGAGAGGTCAGCCATTCTC,

TUBB3 Forward: TCAGCGTCTACTACAACGAGGC,

TUBB3 Reverse: GCCTGAAGAGATGTCCAAAGGC,

Cyclin B1 Forward: GACCTGTGTCAGGCTTTCTCTG,

Cyclin B1 Reverse: GGTATTTTGGTCTGACTGCTTGC,

Cyclin E Forward: TGTGTCCTGGATGTTGACTGCC,

Cyclin E Reverse: CTCTATG TCGCACCACTGATACC,

CDK1 Forward: GGAAACCAGGAAGCCTAGCATC,

CDK1 Reverse: CTCTATGTCGCACCACTGATACC,

CDK2 Forward: ATGGATGCCTCTGCTCTCACTG,

CDK2 Reverse: CCCGATGAGAATGGCAGAAAGC,

CDKN1A Forward: AGGTGGACCTGGAGACTCTCAG,

CDKN1A Reverse: TCCTCTTGGAGAAGATCAGCCG,

β-actin Forward: GGATGCAGAAGGAGATCACTG,

β-actin Reverse: TCCACACGGAGTACTTG

GAPDH or β-actin was used as the housekeeping gene and the target gene expression as quantified by Real-time PCR Detector (CFX Connect, Bio-rad, CA, USA) and analyzed using the Δct power method.

### Flow cytometry and fluorescence image

The cancer cells (A549, AGS, HCT116, HepG2, MCF-7, and MDA-MB-435s) were stained TiNIR (100 nM, ex: 805 nm, em: 825 nm; Merak, Numbrecht, Germany, and Senprobe, Kyngbuk, Republic of Korea) for 30 min after being washed with phosphate-buffered saline (PBS) and destained by medium for 30 min. TiNIR signals (channel: allophycocyanin-Cy7) were detected by flow cytometry (FACSCanto II, BD Biosciences, San Jose, USA). Data analysis was performed using FlowJo software ver. 10.8.1 (TreeStar, OR, USA). For analysis of fluorescence images, various cells (A549, AGS, HCT116, HepG2, MCF-7, and MDA-MB-435s) were prepared appropriately in a glass bottom 96-well plate (Thermo, MO, USA). Cells were stained with TiNIR (1 μM) for 30 min after being washed with PBS and stained with Hoechst 33342 (Thermo, MO, USA) for 10 min. The images were captured with an EVOS M7000 (Thermo, MO, USA) and acquired with a ×40 air objective [10,18].

### Transient transfection with siRNA-HO2

A549 were inoculated into 24-well plates and cultured for 24 h. Then, cells were transfected with siRNA (small interfering ribonucleic acid)-HO2 (30 to 100 pmol, Thrmo, MO, USA) using Lipofectamine 2000 (Invitrogen, CA, USA). Knockdown efficiency was checked by qPCR. The siRNA sequence is as follows: FW: CGGCACUUUACUUCACAUA, RW: GGAGAACCAAAUGAGAAUG.

### Migration and single cell tracking

Cells were seeded in 24-well plates (2.5 × 10^5^/well) and incubated for 24 h. The cells were scratch-wounded and treated at various concentrations of TiNIR or siRNA-HO2 for 40 to 48 h. For the duration of imaging, cells were maintained at 37 °C supplemented with 5% CO_2_. All images were acquired via a ×20 air objective of EVOS M7000. Data analysis was using Celleste Image analysis software ver. 5.0 (Thermo, MO, USA).

Migration was determined as follows: Migration (%) = migrated cell surface area/total surface.

Single cell tracking was analyzed using the manual tracking feature on Celleste ver. 5.0. This analysis was used to record the point of the tangibly identified middle of the cell of target in each image of the sequence. Image sequences (*n* = 12) were analyzed per control and TiNIR-treated condition.

### Proliferation assay

Cells were seeded in triplicate in a 96-well plate (2.5 × 10^4^/well) and incubated for 24 h. The cells were treated at various concentrations of TiNIR for 24 h. CCK-8 reagents (Cell counting kit-8, Dojindo, Kumamoto, Japan) were used to assess cell proliferation. Optical density was quantified by a microplate reader (Versa max, Molecular Devices, CA, USA). 

### 3D ODT imaging

Three-dimensional optical diffraction tomography (3D ODT) images of live A549 cells were obtained by HT-X1 (Tomocube, Daejeon, Republic of Korea), a low-coherence holotomography imaging system with reduced speckle noise. In detail, A549 cells were seeded in a 24-well glass-bottom plate (Cellvis, CA, USA) and maintained in RPMI 1640 (Gibco, MA, USA) supplemented with 10% FBS (Gibco, MA, USA) and 1% penicillin/streptomycin (Gibco, MA, USA) for 24 h. Subsequently, the dishes were washed with Hanks' balanced salt solution (Gibco, MA, USA) and replaced with fresh medium containing either TiNIR (1 μM) or dimethyl sulfoxide. HT-X1 adapts a motorized ×40 numerical aperture 0.95 objective lens (UPLXAPO40X, Olympus, Tokyo, Japan), combining with a 450-nm light-emitting diode illumination module integrated with a digital micromirror device at the pupil plane and a custom-designed condenser lens (numerical aperture = 0.72, working distance = 30 mm). A stage-top incubator (STXG-WSKMXA22BE, TOKAI HIT, Shizuoka, Japan) was integrated into the stage of HT-X1 to maintain 37 °C and 5% CO_2_ conditions. For time-lapse imaging, tens of certain points were chosen to be recorded every 30 min for 24 h. The optimal focus of each point was adjusted by an autofocus system (laser wavelength: 780 nm, range: +100 to −300 μm) digitally controlled by the operating software TomoStudio X (Tomocube, Daejeon, Republic of Korea). To minimize missing cells on the field of view (FOV) during time-lapse imaging, we set FOV as 218 μm × 165 μm, which is the maximum FOV of HT-X1. 3D refractive index (RI) tomograms of the cells with 2D RI distribution were reconstructed by TomoAnalysis (Tomocube, Daejeon, Republic of Korea). For tracing movements of cells in time-lapse imaging, the movement of center positions in each nucleus was analyzed using TomoAnalysis (Tomocube, Daejeon, Republic of Korea).

### Invasion assay

Matrigel-coated chambers were used for the determination of cell migration and invasion, respectively. A549 (2 × 10^5^/well, 24-well plate) with treated various concentrations of TiNIR was added to the upper chambers, and the medium was added to the lower chambers. After 40 h, the migrated and invasive cells were immobilized using 4% formaldehyde and stained with crystal violet (Abcam, MA, USA). Images of the cells with the signal of the crystal violet were acquired via a ×10 air objective. The images were analyzed using Celleste Image analysis software ver. 5.0 (Thermo, MO, USA).

### Next-generation sequencing

Total RNA concentration was calculated by Quant-IT RiboGreen (Thermo, MO, USA). To assess the integrity of the total RNA, samples are run on the TapeStation RNA screentape (Agilent, CA, USA). Only high-quality RNA preparations, with RNA integrity number greater than 7.0, were used for RNA library construction. A library was independently prepared with 1 μg of total RNA for each sample by Illumina TruSeq Stranded mRNA Sample Prep Kit (Illumina, CA, USA). The first step in the workflow involves purifying the poly-A containing mRNA molecules using poly-T-attached magnetic beads. Following purification, the mRNA is fragmented into small pieces using divalent cations under elevated temperature. The cleaved RNA fragments are copied into first-strand cDNA using SuperScript II reverse transcriptase (Thermo, MO, USA) and random primers. This is followed by second-strand cDNA synthesis using DNA polymerase I, RNase H, and deoxyuridine triphosphate. These cDNA fragments then go through an end-repair process, the addition of a single “A” base, and then ligation of the adapters. The products are then purified and enriched with PCR to create the final cDNA library. The libraries were quantified using KAPA Library Quantification kits for Illumina Sequencing platforms according to the qPCR Quantification Protocol Guide (KAPA BIOSYSTEMS, MA, USA) and qualified using the TapeStation D1000 ScreenTape (Agilent, CA, USA). Indexed libraries were then submitted to an Illumina NovaSeq (Illumina, CA, USA), and paired-end (2 × 100 bp) sequencing was performed by Macrogen Incorporated. Heat map and similarity matrix were generated at the MORPHEUS (https://software.broadingstitute.ort/morpheus/) using fragments per kilobase of transcript per million mapped reads values. Then, protein–protein interaction (PPI) network was obtained through the online tool (Search Tools for the Retrieval of Interacting Protein; version 11.5; https://string-db.org) string to differentially expressed genes (Control vs TiNIR, siRNA-HO2).

### Western blot assay

Cells were washed twice with PBS and lysed on ice for 30 min in RIPA lysis buffer (Thermo, MO, USA) supplemented with phosphatase and protease inhibitor cocktail (Thermo, MO, USA). The protein concentration was measured using a bicinchoninic acid assay kit (Thermo, MO, USA). Subsequently, the 4 to 12% Bis-Tris plus gels-separated proteins were transferred to nitrocellulose membranes that were then separately incubated with indicated antibodies. Protein bands were quantified of Amersham Image Quant 800 (GE HealthCare, IL, USA) and analyzed using ImageJ [10].

### Measurement of ROS formation

ROS was detected using a DCFDA/H2DCFDA-Cellular ROS Assay Kit, which was processed according to the manufacturer’s instructions. A549 cells were seeded in a glass-bottom 96-well plate (2.5 × 10^4^/well) and incubated for 24 h. The cells were treated with TiNIR (1 μM) for 2 h and washed with PBS. The cell was stained with DCFDA (dichlorodihydrofluorescein diacetate) at 37 °C for 30 min and washed with PBS. Then, it was stained with Hoechst 33342 (1 μg/ml) and washed with PBS. Image was captured using EVOS M7000 and acquired with a ×40 air objective [10].

### Cell cycle assay

A549 (1 × 10^5^/well, 6-well plate) were treated at various concentrations of TiNIR 12 h. After harvest, cells were washed with PBS and fixed with 70% EtOH for 30 min. Fixed cells were washed with PBS and stained with FxCycle PI/RNase staining Buffer (Thermo, MO, USA). The stained cells were quantified by flow cytometry (FACSCanto II, BD Biosciences, CA, USA). Data analyses were performed using FlowJo software ver. 10.8.1 [10].

### Cytoskeleton staining

For analysis of microtubule, A549 was prepared appropriately in a glass-bottom 96-well plate (Thermo, MO, USA). Cells were treated with TiNIR (0.1, 0.3, and 1 μM) and microtubule stains (ViaFluor 488, Biotium, Fremont, USA) for 30 min after being washed with PBS and stained with Hoechst 33342 (Thermo, MO, USA) for 10 min. For analysis of filamentous actin (F-actin), cells were washed twice in PBS and were fixed in 4% formaldehyde for 15 min followed by 0.2% Triton X-100 for 15 min at room temperature. After blocking of nonspecific binding sites in 4% BSA for 30 min, cells were stained with phalloidin for 30 min at room temperature. At last, nuclear was stained with Hoechst 33342 (Thermo, MO, USA) for 10 min. The images were captured with an EVOS M7000 (Thermo, MO, USA) and acquired with a ×40 air objective. F-actin, microtubule, and nuclei, fluorescence means were calculated using ImageJ. F-actin and microtubule were determined as follows: Mean (%) = F-actin or microtubule fluorescence mean/nuclei fluorescence mean.

### Therapeutic study

The animal study was performed under the approval of the Sunchon National University Institutional Animal Care and Use Committee (SCNU IACUC-2023-4). BALB/c nude mice (6 to 8 wk, CAnN. Cg-Foxninu/CrlOri) were purchased from Orient-bio (Seong-nam, Republic of Korea). The animals were housed in a controlled environment (22 ± 2 °C and humidity 50% ± 5%) in polycarbonate cages and fed a standard animal diet with water. A549 cells (2 × 10^6^/100 μl) were subcutaneously injected into the flank site of nude mice. From week 8, after the postinjection of A549 cells, mice were operated to remove the tumor. From day 1, after surgeries on tumor, TiNIR (100 μM, 100 μl/10 g) was injected into the tail vein 3 times a week until week 8. The vehicle group was injected with the same volume of PBS. Mice were injected TiNIR (100 μM, 100 μl/10 g) into the tail vein 1 d before the achievement of imaging. The TiNIR signal was detected on the IR long channel (836BP46 nm) using Amersham Image Quant 800 (GE HealthCare, IL, USA) in the body and lung. This signal was analyzed using ImageJ software.

### Tissue image

The harvested lungs were washed with PBS to remove the blood and frozen in the optimal cutting temperature (Surgipath FSC 22, Leica, Wetzla, Germany). Cryosectioned tissue (14 μm) was prepared using a freezing microtome (Amos Scientific, Melbourne, AU). The section was washed with PBS to remove optimal cutting temperature. The section was stained using a hematoxylin and eosin stain kit (Abcam, MA, USA) according to the manufacturer’s instructions. The fluorescence image was stained diamond antifade mount with 4′,6-diamidino-2-phenylindole (Thermo, MO, USA). The image was obtained by EVOS M7000 and ×20 and ×40 air objectives. The image was analyzed using ImageJ and Celleste image analysis software ver. 5.0 (Thermo, MO, USA) [18].

### Statistical analyses

Statistical analysis was performed by Student *t* test for comparison between 2 groups or 1-way analysis of variance with Tukey’s multiple evaluations for comparison between more than 2 groups. Statistical calculations for proliferation, invasion, migration, mRNA levels, protein levels, cell cycle, and lung region of interest analysis were performed using SPSS ver. 27 (SPSS, IL, USA), and those for holotomography imaging were performed using Prism 9.1 (GraphPad, La Jolla, CA). *P* < 0.05 was considered to indicate a statistically significant difference. All values are expressed as means ± standard deviation (SD) except Fig. 3B (means ± standard error of the mean [SEM]).

## Results

### Identification of HO2 as a biomarker for metastatic cancer therapy

**Fig. 1.**
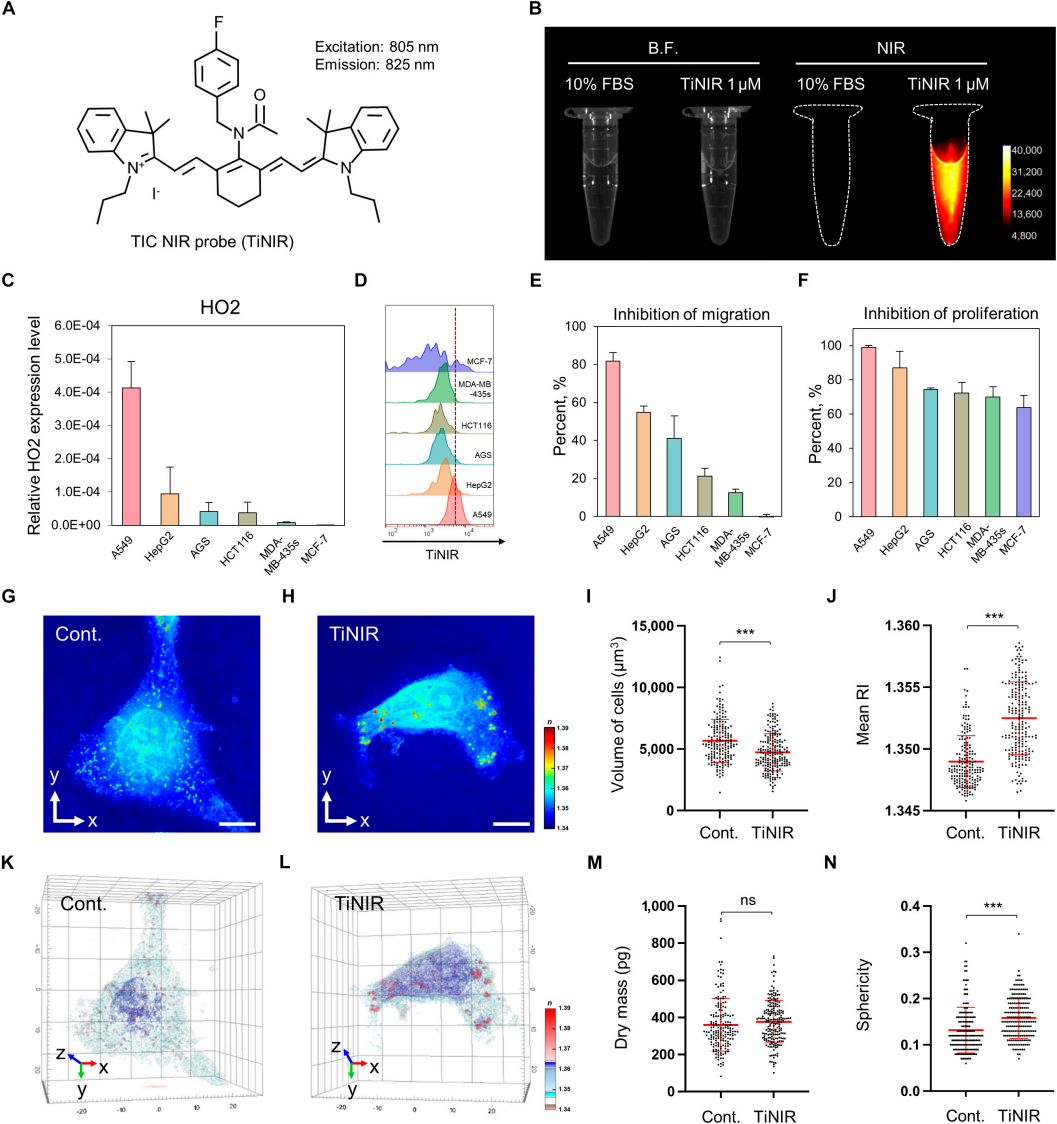
Correlation between expression of HO2 and behavior of cancer cells. Structure (A) and fluorescence emission (B) of TiNIR. The photographs showed PBS and TiNIR with 10% FBS under NIR. (C) HO2 mRNA expression level in various cells (A549, HepG2, AGS, HCT116, MDA-MB-435s, and MCF-7) quantified by qPCR. (D) TiNIR staining pattern in the 6 kinds of a cancer cell line. TiNIR signal was detected by flow cytometry. Mean fluorescence intensities ± SD of 3 independent experiments are shown. (E) The graph presents the inhibitory effect of TiNIR (1 μM) on cancer cell migration (Migration [%] = migrated cell surface area/total surface). (F) Proliferation of cancer cells was measured after treatment of TiNIR (1 μM) for 48 h. (E to H) ODT for label-free 3D imaging using RI mapping in A549 cells after treatment of TiNIR (1 μM). (G and H) Jet scale images of A549 cells either treated with medium only or medium containing TiNIR. Scale bars: 10 μm. (I and J) Three-dimensional reconstruction of images in (G) and (H), respectively. 2D representations of the refractory index are located on the side of the figures. (K to N) Quantitative analysis of 4 characteristics of live A549 cells. Data are shown means ± SD (*n* = 178 and 201 for control and TiNIR, respectively; *P* values were determined by unpaired *t* test *P < 0.05, **P < 0.01, ***P < 0.001). B.F., bright field.

To investigate the bioavailability and potential of HO2 as a therapeutic biomarker of metastatic cancer, we measured HO2 expression levels across various cancer cell lines, including A549, HepG2, AGS, HCT116, MDA-MB-435s, and MCF-7 cells. Among these, A549 cells exhibited the highest level of HO2 expression (HO2 expression levels: A549 > HepG2 > AGS > HCT116 > MDA-MB-435s > MCF-7) (Fig. 1C). To validate the correlation between HO2 expression levels and TiNIR, previously developed as an HO2 tracker and inhibitor, we applied TiNIR in 6 cell lines and quantitatively analyzed the result using flow cytometry. As a fluorescent compound, TiNIR exhibited a stable high fluorescence signal (IR long: 836BP46 nm) under 775-nm excitation in 10% FBS PBS (Fig. 1A and B). Consistent with mRNA expression patterns, TiNIR-stained cancer cell lines exhibited HO2 expression levels in a dependent manner of TiNIR intensity; A549 > HepG2 > MDA-MB-435s > AGS > HCT116 > MCF-7 (Fig. 1D). This trend was also observed through fluorescence microscopy (Fig. S1A). As a second step, we assessed the inhibitory effects of TiNIR on the migration of the cancer cell lines to verify the bioavailability of HO2 for cancer therapy, given its therapeutic effects previously validated on TICs. TiNIR treatment strongly suppressed migration of cancer cells in an HO2-expression-dependent manner (Fig. 1E and Fig. S1B). Furthermore, the proliferation of each cancer cell line was markedly inhibited by TiNIR in an HO2-expression-dependent manner (Fig. 1F and Fig. S1C). Notably, TiNIR did not induce cell death compared to the starting point cell survival value (Fig. S1C). In a third line of investigation, to observe the effects of TiNIR on live A549 cells, we leveraged the advantages of 3D holotomography, a technique verified in previous studies [19–21]. Clear differences in shape were observed between the control and TiNIR-treated live A549 cells (Fig. 1G to J). Quantitative analysis revealed smaller volumes and higher values of mean RI and sphericity in TiNIR-treated live A549 cells compared to the control groups (Fig. 1K to N). These results strongly suggest that HO2 could serve as a valuable therapeutic biomarker, given its close association with the observed effects.

### Regulatory effect of HO2 on migrating lung cancer cells

**Fig. 2.**
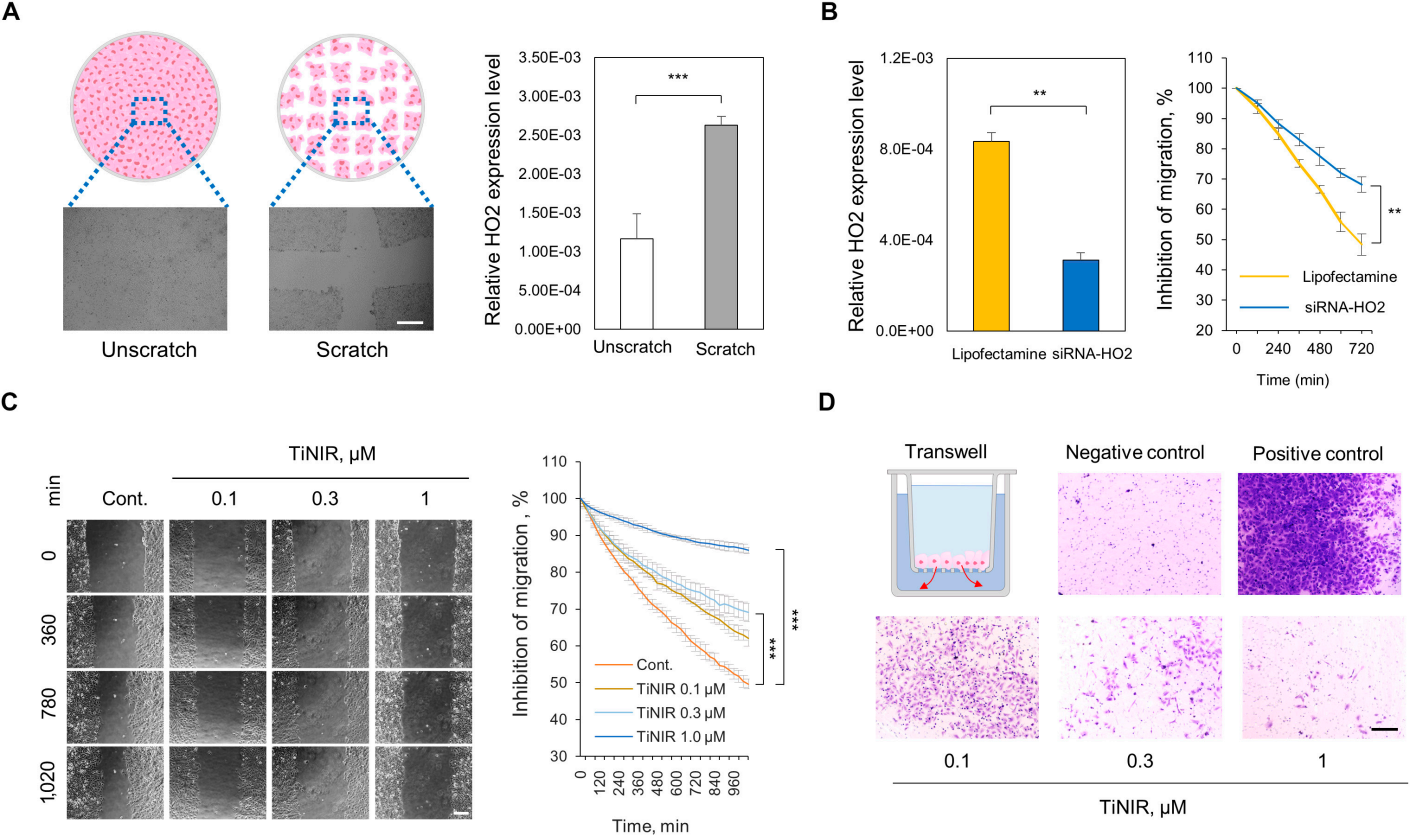
HO2 is a key player in cancer cell migration and invasion. (A) Differential expression of HO2 mRNA according to the state of cells. Scheme of experiment (left). HO2 mRNA level was confirmed by qPCR in the different cell conditions (right). Scale bars: 500 μm. (B) Correlation between expression of HO2 and migration of cells. A549 cells were treated with siRNA-HO2 (30 to 60 ρmol) for 48 h. HO2 mRNA level was confirmed by qPCR. The graph represents the percent (%) of inhibition on the cell migration at the scratch area. Data showed as means ± SD (*n* = 4, *P* values were determined by 1-way Tukey’s test **P < 0.01). (C) Migration was measured in 30-min intervals for 40 to 48 h after treatment with various concentrations of TiNIR. Representative microscopy images (EVOS M7000, ×20 air objective) of migration at the 4 time points. Scale bars: 200 μm (left). The graph represents the percent (%) of inhibition on the cell migration at the scratch area (right). Images were analyzed using Celleste Image analysis software ver. 5.0 and are shown as means ± SD (*n* = 6, *P* values were determined by 1-way Tukey’s test *P < 0.05, **P < 0.01, ***P < 0.001). (D) Transwell analysis for invasion of cancer cells. To check the invasion, the cell was seeded in Matrigel-coated Transwell plates and treated with TiNIR for 40 h. Cell invasion was measured by crystal violet staining and imaged using the EVOS M7000 imaging system. Scale bars: 200 μm.

Our study confirmed that the HO2 inhibitor effectively inhibited cancer cell proliferation and migration depending on HO2 expression levels in each cancer cell line. These results highlight the potential of HO2 as a biomarker. To validate the therapeutic potential of HO2 inhibition, we selected A549 cells that exhibit high levels of HO2 expression. In investigating the dynamics of HO2 expression during different stages of cell culture, we employed a scratched cell culture model (Fig. 2A, left). Our results revealed that dynamically moving cells exhibited significantly higher levels of HO2 expression compared to stationary cells (Fig. 2A, right). To confirm a direct correlation between HO2 expression and migration of cancer cells, we conducted a siRNA-mediated knockdown of HO2 in A549 cells. Our findings indicated that HO2 knockdown significantly diminished the migratory ability of A549 cells (Fig. 2B and Fig. S2). To further investigate the function of HO2 in cancer cell migration and invasion, TiNIR, proven previously for its inhibitory effect on HO2, was applied to A549 cells at various concentrations. Notably, our results indicated that TiNIR suppressed cancer cell migration in a concentration-dependent manner (Fig. 2C and Movies S1 and S2). Furthermore, we assessed the inhibitory effect of TiNIR on cancer cell invasion using a Transwell assay, revealing significant inhibition of cancer cell invasion in a concentration-dependent manner (Fig. 2D). These findings demonstrate that the inhibition of HO2 effectively suppresses both cancer cell migration and invasion, providing compelling evidence for the therapeutic potential of HO2 as a target in cancer.

### Effect of HO2 inhibitor on the behavior of single lung cancer cells

**Fig. 3.**
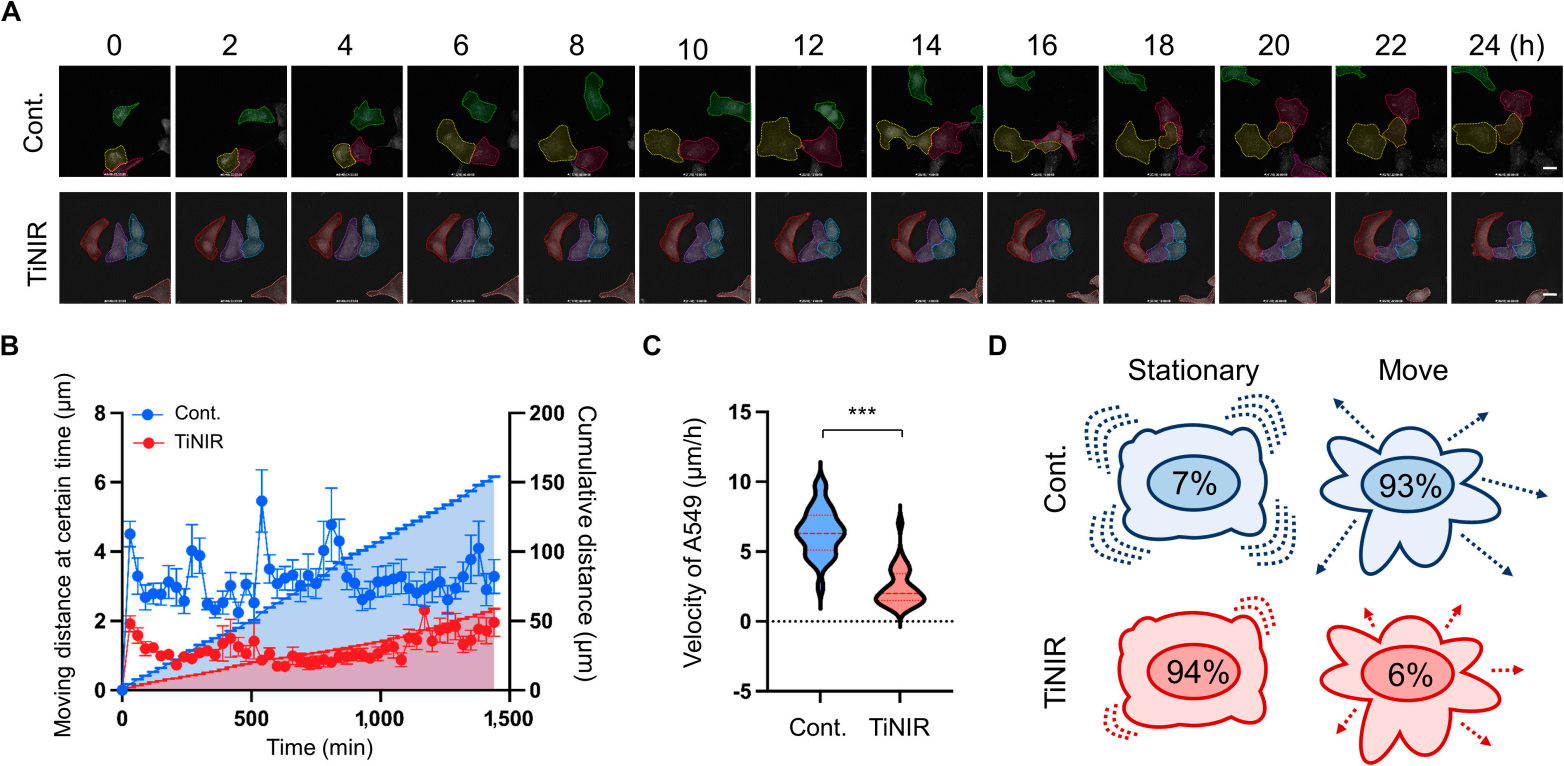
Inhibitory effects for single-cell movement by the HO2 inhibitor (TiNIR). (A) Time-lapse imaging of migration in live control A549 cells (top) and TiNIR-treated A549 cells (bottom). Few cells in control groups are marked with dotted lines. Note that none of the TiNIR-treated A549 cells moved outside FOV. Scale bars: 20 μm. (B) Moving distance at a certain time (dot plot, left Y axis) and cumulative distance (integrated area, right Y axis) of 2 groups. Data are shown as means ± SEM (*n* = 30 and 31 for control and TiNIR, respectively). (C) Average velocity of control and TiNIR-treated A549 cells. Violin plot representing the distribution of the number of each group. The thicker the region size is, the more cells are located. Each dotted line represents the bottom, first quartile, middle, median, top, and third quartile. *P* values were determined by unpaired *t* test *P < 0.05, **P < 0.01, ***P < 0.001. (D) The ratio of “Stationary” and “Move” for each group. As written in the main text, we divided these into whether (Move) or not (Stationary) the cell migrates more than 100 μm.

To further investigate the inhibitory effect of HO2 at the single-cell level on the migration of live A549 cells, we meticulously tracked their movement under a stable observation environment for 24 h or more. As anticipated, the cells treated with TiNIR exhibited significantly reduced migration compared to the nontreated control cells (Fig. 3A to C and Movies S3 and S4). When we divided cells into 2 groups, “Stationary” (movement less than 100 μm in 24 h) and “Move” (movement more than 100 μm in 24 h), the composition of “Move” was higher in the control group, a trend reversed in the TiNIR-treated group (Fig. 3D). This observation underscores the effective hindrance of migratory capacity by inhibition of HO2 with TiNIR treatment in A549 cells compared to untreated controls.

### Regulation of metastatic characteristics by controlling AKT and cJUN signaling via inhibition of HO2

**Fig. 4.**
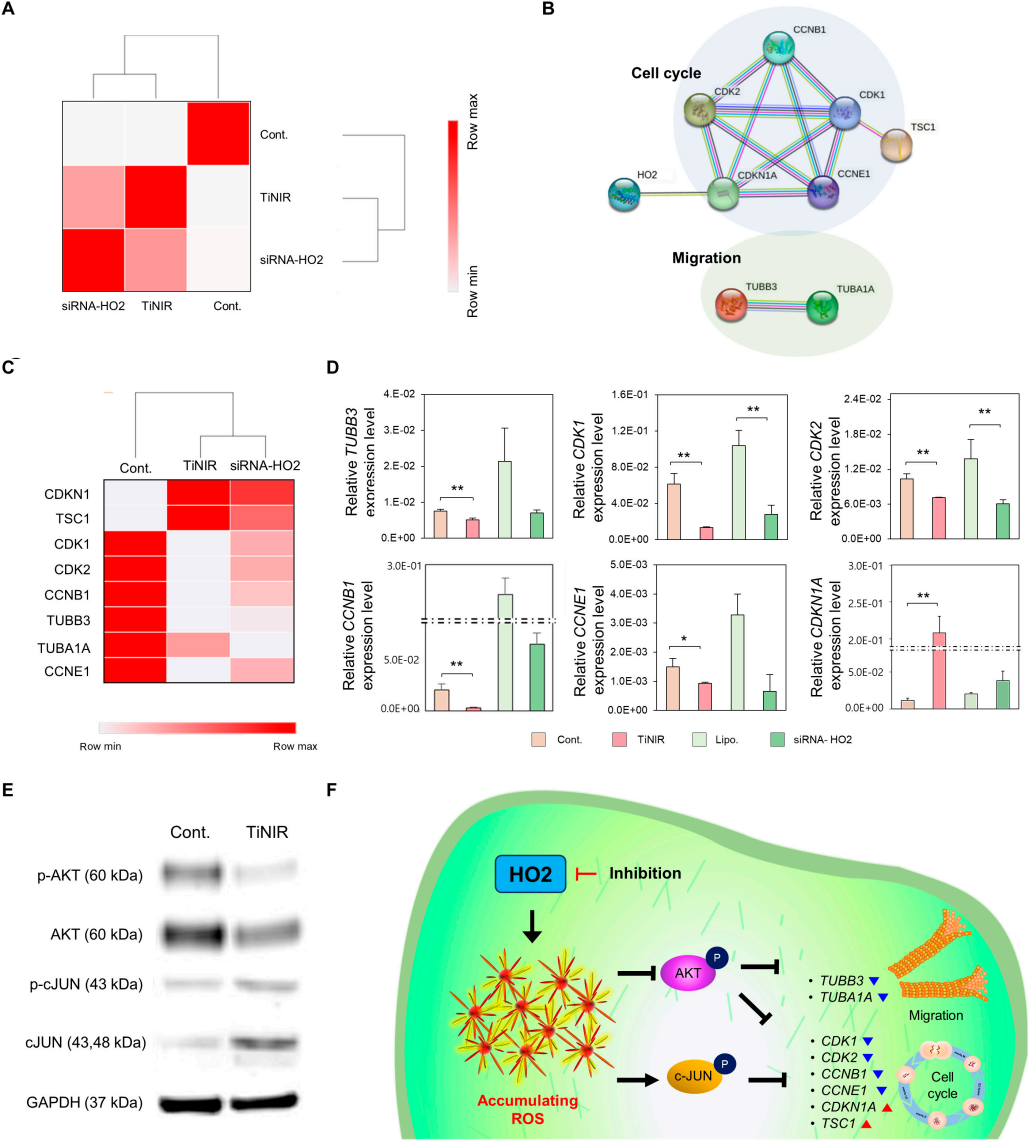
The impact of HO2 inhibition on the AKT/cJUN pathway and its downstream signaling. A549 were treated with TiNIR or siRNA-HO2 for 48 h to confirm the gene expression relationship. (A) Similarity matrix of the fragments per kilobase of transcript per million mapped reads value from RNA-seq analysis. The list of 6,605 genes was extracted from the RNA-seq list, which was selected by 15% up-regulated and 15% down-regulated mRNA in the TiNIR-treated or siRNA-HO2-treated A549 compared to control cells. (B) Confirmation of relative proteins with HO2 by PPI analysis. Eight kinds of proteins were identified using PPI analysis (STRING; version 11.5; https://stringdb.org). (C) The heatmap of mRNA expression of 8 proteins. Data was obtained from RNA-seq (Control vs. TiNIR vs. siRNA-HO2). (D) Quantitative assessment of representative HO2-associated genes. The mRNA levels were analyzed by qPCR after treatment of TiNIR and siRNA-HO2. Data are shown means ± SD (*n* = 3, *P* values were determined by Student *t* test *P < 0.05). (E) Phosphorylation of upstream proteins, AKT and cJUN. The phosphorylation of AKT and cJUN were analyzed by western blot after treatment of TiNIR (1 μM, 24 h). (F) Schematic representation of regulating AKT and cJUN signaling by inhibiting HO2 (Down-regulation: ▼, Up-regulation: ▲).

The migration capacity of cancer cells is widely recognized as a crucial aspect of cancer metastasis. To elucidate the role of HO2 in cancer metastasis, we conducted a comprehensive analysis of RNA expression levels in normal cells versus HO2 knockdown cells (Fig. S3A) and TiNIR-treated cells, using RNA sequencing (RNA-seq) analysis. Subsequently, we identified genes that showed a 15% difference (up- and down-regulated genes) in expression levels in both the TiNIR and siRNA-HO2 groups compared to the control group, totaling 6,605 genes (Fig. S3B). A similarity matrix, which was to assess the similarities among the selected genes, indicated that the expression patterns of the 6,605 genes were concordant between the TiNIR and siRNA-HO2 groups (Fig. 4A). Both siRNA-based knockdown (HO2-siRNA) and TiNIR-based inhibition of HO2 have demonstrated a reduction in the proliferation and metastatic activity of cancer cells. To unravel the underlying mechanisms, we extracted proliferation- and migration-associated genes from the 6,605 genes and analyzed their protein networks through the PPI database (Fig. 4D). Our analysis revealed that both HO2-siRNA and TiNIR treatment similarly regulated the expression of key cell cycle regulators [22,23], including *CDK1*, *CDK2*, *CCNB1*, *CCNE2*, *CDKN1A*, and *TSC1*, as well as critical factors for cell migration [24–26], such as *TUBA1A* and *TUBB3* (Fig. 4C). We validated these changes in representative genes, including the expression of migration-related *TUBB3* and several cell-cycle-related genes (*CDK1*, *CDK2*, *CCNB1*, *CCNE1*, and *CDKN1A*) (Fig. 4D).

Previous studies have demonstrated that p-AKT induces microtubule stabilization, playing a crucial role in cell growth and migration [27,28]. To further elucidate the intricate connection between HO2, the cell cycle, and migration, we scrutinized AKT phosphorylation in A549 cells. Our investigation revealed a decrease in AKT expression in the TiNIR-treated group compared to the control group, accompanied by a reduction in phosphorylation (Fig. 4E). Furthermore, we noted an elevated level of protein expression and phosphorylation of cJUN, a transcription factor for p21 (gene name: *CDKN1A*) in the TiNIR-treated group (Fig. 4E). This aligns with our observation of increased CDKN1A in response to TiNIR treatment in A549 cells. The PPI analysis revealed a close association among HO2, AKT, cJUN, and the other key regulatory genes in cell cycle and migration (Fig. S3D). These findings strongly suggest that HO2 inhibition exerts a suppressive effect on cancer cell growth and migration capacity by modulating both the cell cycle and migration pathways through the regulation of AKT and cJUN expression and phosphorylation (Fig. 4F).

### Changes of cell cycle and cytoskeleton in the lung cancer cells treatment of HO2 inhibitor

**Fig. 5.**
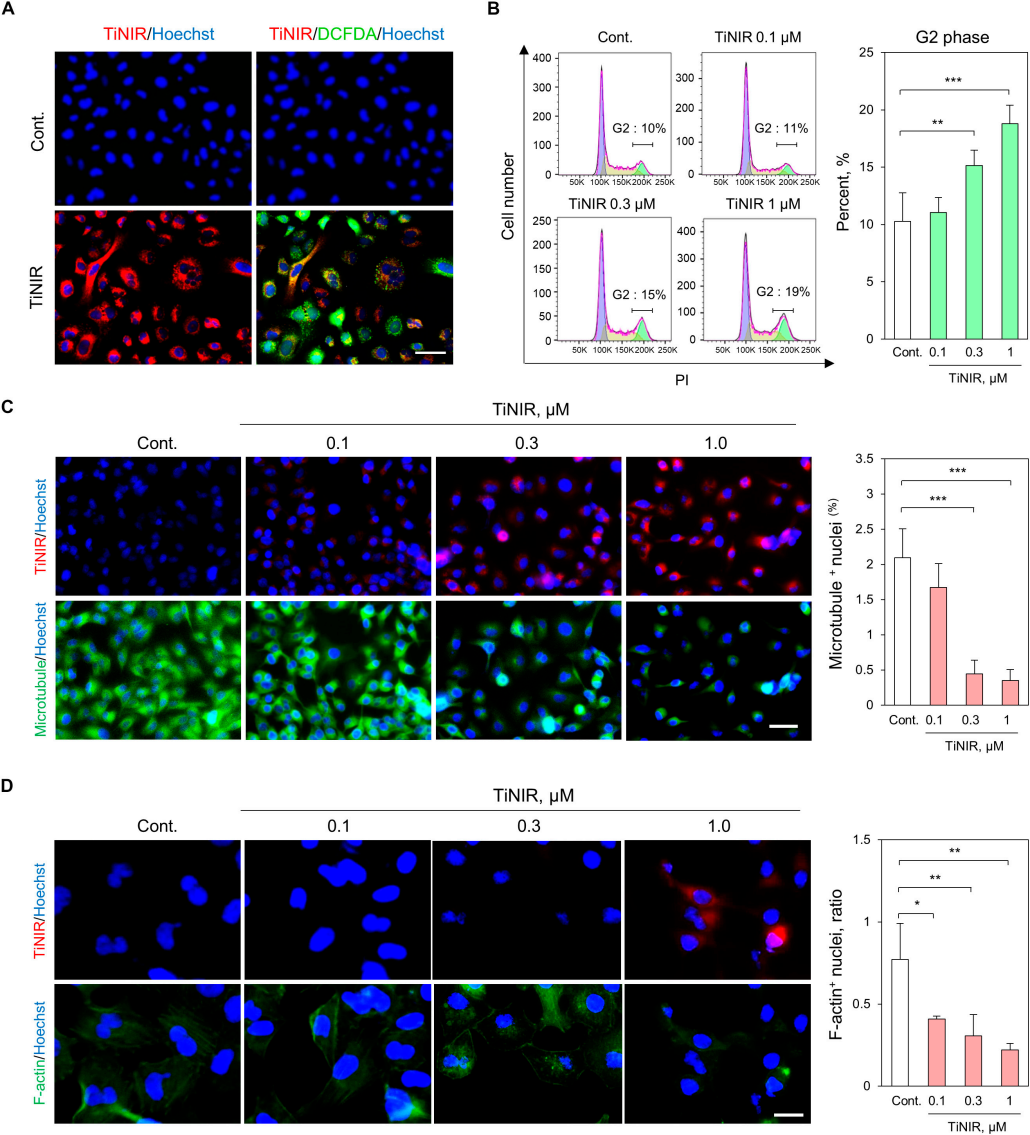
Inhibiting HO2 induces the arrest of G2/M and dysfunction of the cytoskeleton in A549. (A) Production of ROS by treating TiNIR in the A549. Cells were treated with TiNIR (1 μM, 2 h) and stained with DCFDA to check ROS level. Nuclei were stained with Hoechst. Images were obtained by fluorescence microscopy (EVOS M7000, ×40 air objective). Scale bars: 50 μm. (B) The cell cycle of TiNIR treated A549. The cell cycle was analyzed by propidium iodide (PI) staining and flow cytometry. The quantitative phases of the cell cycle were analyzed using the Watson pragmatic method using FlowJo software ver. 10.8.1. Data are shown means ± SD (*n* = 3, *P* values were determined by 1-way Tukey,s test *P < 0.05, **P < 0.01, ***P < 0.001). Effect of TiNIR on the reorganization of the cytoskeleton in A549. Cells were stained with a (C) microtubule (green, scale bars: 40 μm) or (D) F-actin (green, scale bars: 20 μm) after treatment of TiNIR. Images were obtained using the EVOS M7000 with ×40 air objective. The graph was shown data on the ratio F-actin/nuclei means ± SD (*n* = 3, *P* values were determined by 1-way Tukey’s test *P < 0.05, **P < 0.01, ***P < 0.001).

We further delved into the HO2 inhibition effect on cell cycle genes at the cellular level. Initially, the inhibition of HO2 by TiNIR led to ROS accumulation in A549 cells (Fig. 5A), a phenomenon also observed in the TIC model by disrupting the biliverdin–bilirubin redox cycle [10]. Building on previous findings that ROS accumulation induces cell cycle arrest in cancer cells [27], we investigated a direct connection between HO2 inhibition and the regulation of the cell cycle and migration. In the cell cycle assays using propidium iodide staining, we observed a G2 phase arrest with the treatment of TiNIR (1 μM) compared to the control, accompanied by a complementary decrease in the S phase correlated with the increase in the G2 phase (Fig. 5B). Cell migration is intricately controlled by dynamic and spatial regulation of the cytoskeleton. Inhibition of HO2 reduced the expression of *TUBA1A* and *TUBB3*, and we tracked the microtubule patterns at the cellular level. The fluorescence image showed a decrease in microtubule growth upon treatment with TiNIR (Fig. 5C). We further confirmed the suppression of p-AKT and TUBB3 expression in the TiNIR-treated cells. p-AKT is known to regulate the assembly of F-actin filaments, enlarging lamellipodia by accumulating F-actin [28]. In the lamellipodia-forming region, cells migrate through microtubule polymerization and depolymerization [29]. Therefore, TiNIR was treated in the cells to investigate the formation of F-actin, and the result showed inhibited F-actin formation upon TiNIR treatment (Fig. 5D). These findings collectively suggest that inhibiting HO2 exerts an impact on G2 phase cell cycle arrest and microtubule formation, thereby intricately regulating cancer cell proliferation and metastasis.

### The therapeutic effect of TiNIR on in vivo metastatic lung cancer

**Fig. 6.**
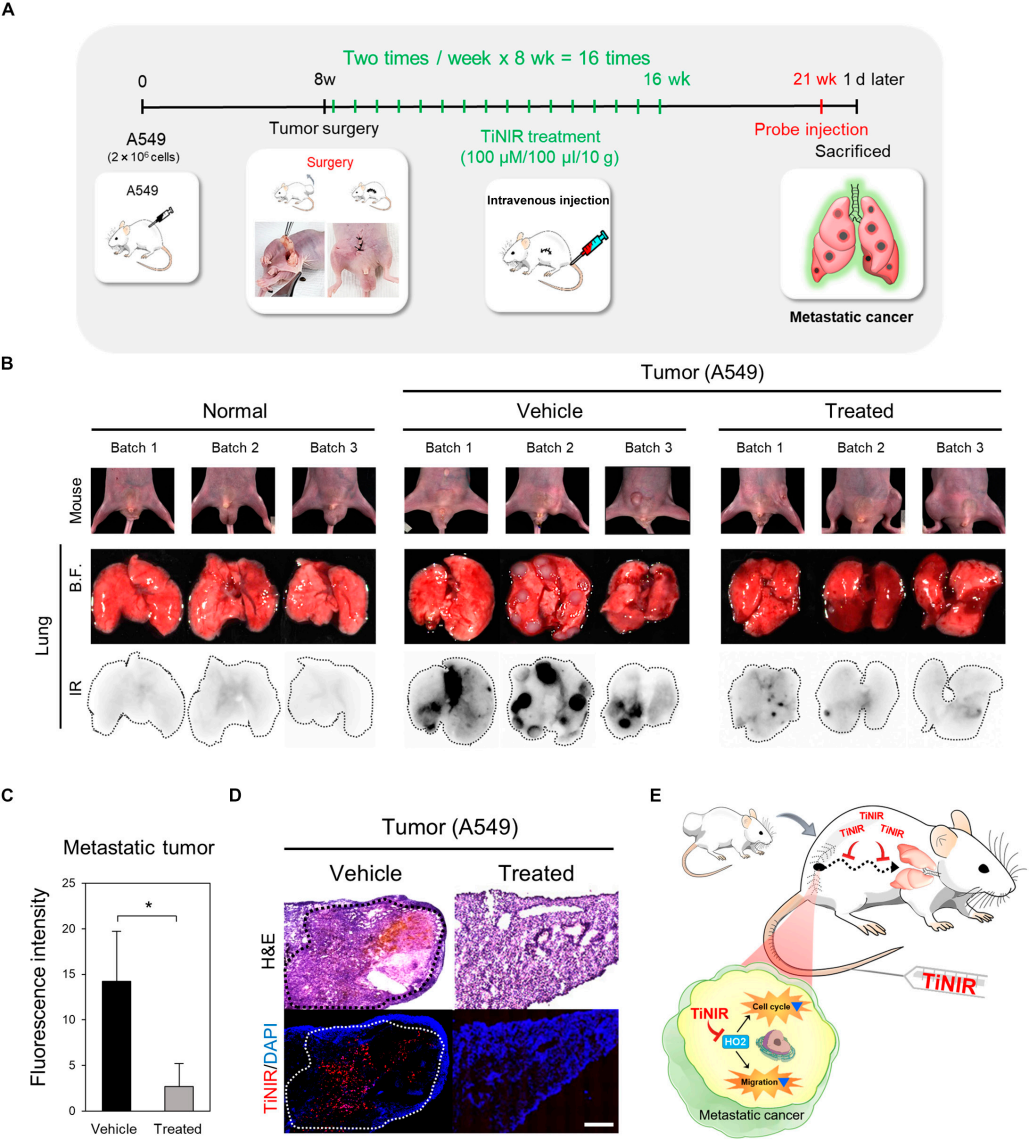
The therapeutic efficacy of HO2 inhibition on in vivo metastasis. (A) Schematic of experimental protocol for metastatic cancer therapy by treating HO2 inhibitor. After 8 weeks postinjection of A549 cells, the tumors were removed by surgery, and TiNIR (100 μM, 100 μl/10 g, HO2 inhibitor) was injected into the tail vein twice a week for 8 weeks. (B) Confirmation of metastatic tumor ex vivo. Three days before tracking the metastatic tumor, the injection of TiNIR was stopped to remove the background signal. Twenty hours before sacrifice, TiNIR (100 μM, 100 μl/10 g) was injected into the mouse tail vein (*n* = 3). After collecting lung tissues, the metastatic tumor signal was detected at the 836BP46-nm wavelength (Amersham Image Quant 800). TiNIR signal was indicated with black. (C) Intensity of TiNIR was quantified by ImageJ software. Data are shown means ± SD (*n* = 3, *P* values were determined by Student *t* test *P < 0.05). (D) Histological analysis of the therapeutic effect of TiNIR in the metastasis lung. The lung tissues were stained by hematoxylin and eosin (H&E) (up) and checked for tumor lesions. Injected TiNIR signals (down) in the metastatic tumor lesions were investigated by fluorescent microscopy (EVOS M7000, ×40 air objective). (E) Schematic representation of HO2 inhibition by TiNIR at the cellular level. Red arrows indicate the inhibition of an HO2 function by TiNIR. DAPI, 4′,6-diamidino-2-phenylindole.

To evaluate the therapeutic potential of targeting HO2 in cancer metastasis, a metastatic cancer model was generated by tumor xenografting achieved by subcutaneous injection of A549 cells in nude mice. Following the surgical removal of subcutaneous tumor chunks under anesthesia at the postinjection of the cells, the mice were allowed to persist for 12 to 13 weeks. To assess the therapeutic effect of HO2 inhibition, we administered TiNIR, as an HO2 inhibitor, intravenously for 8 weeks (twice a week, 100 μM, 100 μl/10 g) (Fig. 6A). One day prior to the sacrifice of the mice, an additional injection of TiNIR was given with the aid of tracking the metastatic tumors. While no tumor nodules were observed in the lungs of normal control mice (normal group; Fig. 6B, left), the lung of the metastatic cancer model mice (vehicle group; Fig. 6B, middle) exhibited robust metastatic tumors, which were visible to the naked eye, accompanied by a distinct fluorescent signal of TiNIR (vehicle group; Fig. 6B, middle). Notably, the generation of metastatic tumors was significantly reduced by treated group (Fig. 6B, right), evident in markedly decreased fluorescence intensity of TINR compared to the vehicle group (Fig. 6C). Additionally, histological sections of the lung tissues clearly illustrated the suppressive effect of the treated group on metastatic cancer generation (Fig. 6D, normal group: Fig. S4), suggesting that HO2 inhibitor holds promise as a potential therapeutic drug for metastasis, particularly in lung cancer (Fig. 6E).

## Discussion

Although HO2 has been suggested as a potential therapeutic biomarker for TICs [10], its functional impacts on cancer therapeutics have not been properly identified. In the present study, we sought to determine the functional implications of HO2 inhibition as a novel therapeutic target of cancer metastasis. We first validated the relationship between the expression level of HO2 and the response to its theragnostic tool, TiNIR, developed for both tracking and inhibiting [10]. Interestingly, the TiNIR treatment showed inhibitory effects on metastatic phenotypes, such as cell proliferation and migration, and those were strongly synchronized with the expression level of HO2 across various cancer cell lines. In addition, we demonstrated that TiNIR could be utilized for visualization and quantitative analysis of HO2 expression with a microscope and flow cytometry, respectively, in various cancer cell lines.

We previously noted from our earlier study conducted in TIC model that HO2 targeting significantly induces ROS accumulation and, as a result, effectively inhibits TIC activity [10], yet downstream pathway has not been elucidated. Through a transcriptome analysis of the metastatic cancer cell model, we unraveled potential connections between HO2 expression and cancer metastasis in this study. HO2 inhibition activated the cJUN signaling pathway while simultaneously suppressing the activation of the AKT signaling pathway. These orchestrated the expression of key genes associated with proliferation and cell migration, including *CDK1*, *CDK2*, *CCNB1*, *CCNE1*, *CDKN1A*, *TSC1*, *TUBA1A*, and *TUBB3*, resulting in showing antimetastatic phenotypes (Fig. 4E). The orchestration of the cell cycle hinges upon the delicate interplay between CDKs and cyclins, orchestrating the sequential progression through G1, S, G2, and M phases. Notably, our study demonstrated a reduction in CDK2, the initiator of the G2 phase, and *CDK1*, the activator of the M and G2 phases, subsequent to the inhibition of HO2.

TUBB3, which was previously suggested as a cancer cell biomarker [24–26], encodes β-tubulin, playing a vital role in various cellular processes, including cell migration and the cell cycle [22,23]. Here, we validated the decreased mRNA levels of TUBB3 following the treatment of cancer cells with TiNIR and siRNA. Based on the research performed by Fink et al. in 2020, we highlight the pivotal role of the cytoskeleton in cellular dynamics, especially in cancer metastasis characterized by lamellipodia, which promotes cell migration and directs cancer cells to secondary sites [29,30]. In particular, the activation of *AKT1* promotes the reorganization of F-actin, which is crucial for lamellipodia formation. In our study, we observed that HO2 inhibition led to the suppression of AKT phosphorylation. This suppression ultimately impeded F-actin formation and the dynamics, consequently hindering cell motility. The intricate genetic regulations identified in this study exerted a profound impact on cytoskeleton dynamics. Our in vitro findings demonstrated that targeting HO2 effectively suppressed both cell proliferation and mobility. Specifically, G2 arrest was induced, and microtubule and microfilament formation were inhibited. Additionally, our further investigation using 3D ODT for validating the physical properties of cells at the cellular level showed that the treatment with TiNIR remarkably induced increased RI and sphericity of cells, indicative of reduced migration and invasion [30–32]. These observations aligned seamlessly with in vivo observations, where the treatment with an HO2 inhibitor led to the suppression of cancer metastasis. The robustness of these findings substantiates our conclusions that targeted HO2 therapy emerges as a potent suppressor of lung metastasis.

In summary, our investigation has demonstrated that regulating HO2 leads to the accumulation of ROS, consequently exerting control over the proliferation and migration of A549 cells through the regulation of AKT and cJUN expression and phosphorylation. In addition, an HO2 inhibitor evidently inhibited metastatic lung cancer progression in a mouse model. In conclusion, our results affirm that targeted HO2 therapy represents a highly promising strategy for impeding both cancer cell migration and proliferation, thus presenting a viable avenue for the treatment of metastatic cancer.

## Data Availability

The datasets used and/or analyzed during the current study are available from the corresponding author on reasonable request.
