# Supplementary material for: Targeting Heme Oxygenase 2 (HO2) with TiNIR, a Theragnostic Approach for Managing Metastatic Non-Small Cell Lung Cancer
Source: Biomater Res. 2024 Apr 25;28:0026. doi: 10.34133/bmr.0026 (PMC11045274; doi:10.34133/bmr.0026)
Supplement: Supplementary 1 — Figs. S1 to S4 Movies S1 to S4 [file bmr.0026.f1.zip › Supplementary Information.docx]

**Supporting Information**

**Targeting Heme Oxygenase 2 (HO2) with TiNIR, a theragnostic approach for managing metastatic non-small cell lung cancer**

Seul-Ki Mun ^1, 2, *^, Hyun Bo Sim ^1, *^, Jae-Hyuk Lee ^3, *^, Hyeongyeong Kim ^1^, Dae-Han Park ^1^, Yong-An Lee ^4^, Ji Yeon Han ^1^, Yu-Jeong Choi ^1^, Jun Sang Son ^1^, Jeongwon Park ^3^, Tae-Hwan Lim ^2^, Sung-Tae Yee ^2^, Young-Tae Chang ^5,6^, Seongsoo Lee ^3, 7 #^, Dong-Jo Chang ^2,#^, and Jong-Jin Kim ^1,#^

^1^ Department of Biomedical Science, Sunchon National University, Suncheon 57922, Republic of Korea.

^2^ College of Pharmacy, Sunchon National University, Suncheon 57922, Republic of Korea.

^3^ Gwangju Center, Korea Basic Science Institute (KBSI), Gwangju 61751, Republic of Korea.

^4^ Genome Institute of Singapore (GIS), Agency for Science, Technology and Research (A*STAR), 60 Biopolis Street, Genome, Singapore 138672, Republic of Singapore.

^5^ School of Interdisciplinary Bioscience and Bioengineering, Pohang University of Science and Technology (POSTECH), Pohang 37673, Republic of Korea.

^6^ Department of Chemistry, Pohang University of Science and Technology (POSTECH), Pohang 37673, Republic of Korea.

^7^ Department of Systems Biotechnology, Chung-Ang University, Anseong 17546, Republic of Korea.

^*^ These authors contributed equally: Seul-Ki Mun, Hyun Bo Sim, Jae-Hyuk Lee

^#^ Corresponding authors: Email addresses: Seongsoo Lee (soolee@kbsi.re.kr), Dong-Jo Chang

**Supplementary Figures**


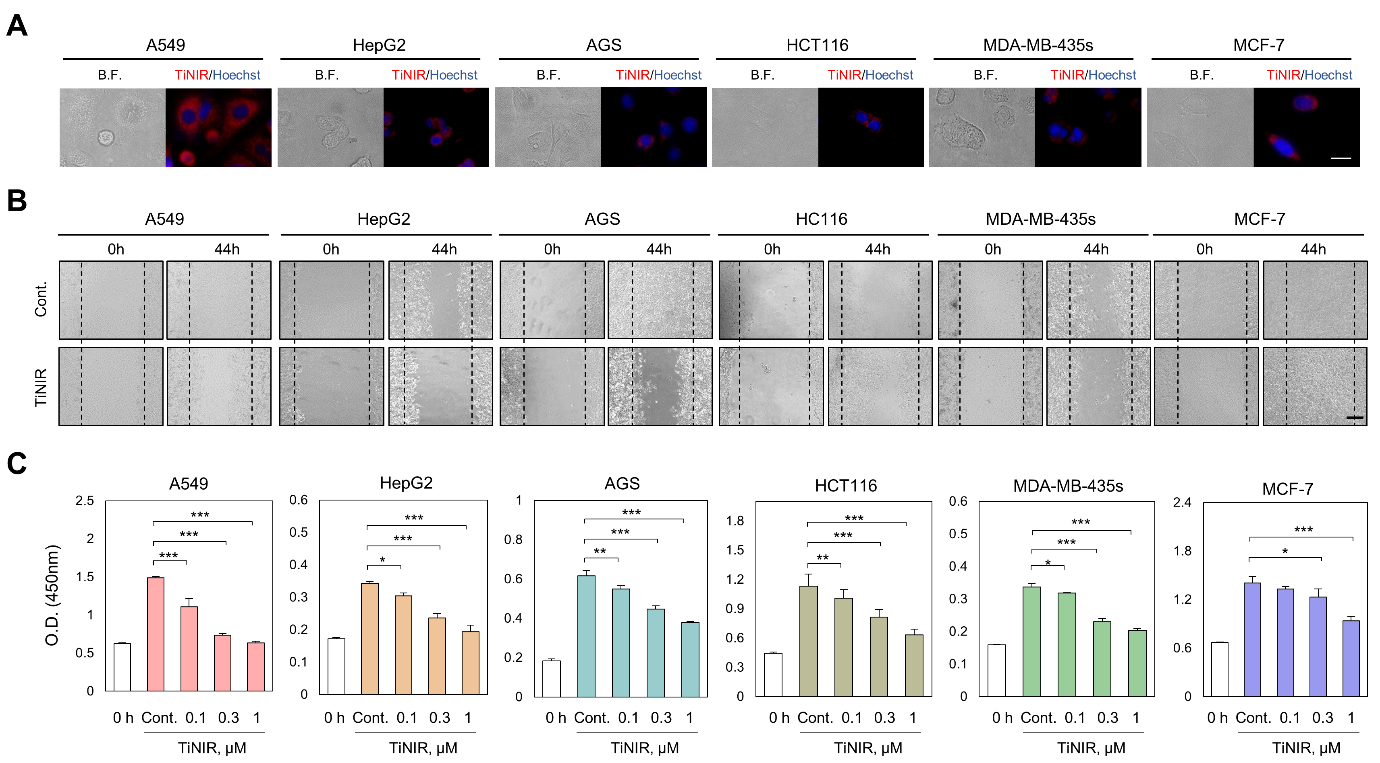


**Figures S1. HO2-dependent response of cancer cells by treatment of HO2 inhibitor, TiNIR.** **A** Fluorescence images of cancer cells were detected by fluorescence microscopy. The cells were stained TiNIR (1 μM) and Hoechst (1 μg/mL) for 1 h. Scale bar: 10 μm. **B** Effect of TiNIR on the migration of cancer cells. The cells were cultured in a 24-well plate, scratched to make a wound, and treated with TiNR (1 μM). Representative images of migration were captured in the area of scratch at the start or end point of the experiment. Scale bars: 200 μm. **C** Proliferation of cancer cells was measured 24 h after TiNIR treatment using CCK-8 (optical density at 450 nm) assay. Data are shown mean ± SD (*p*-values determined by one-way Tukey's test, n=3, **p*<0.05, ***p*<0.01, ****p*<0.001).


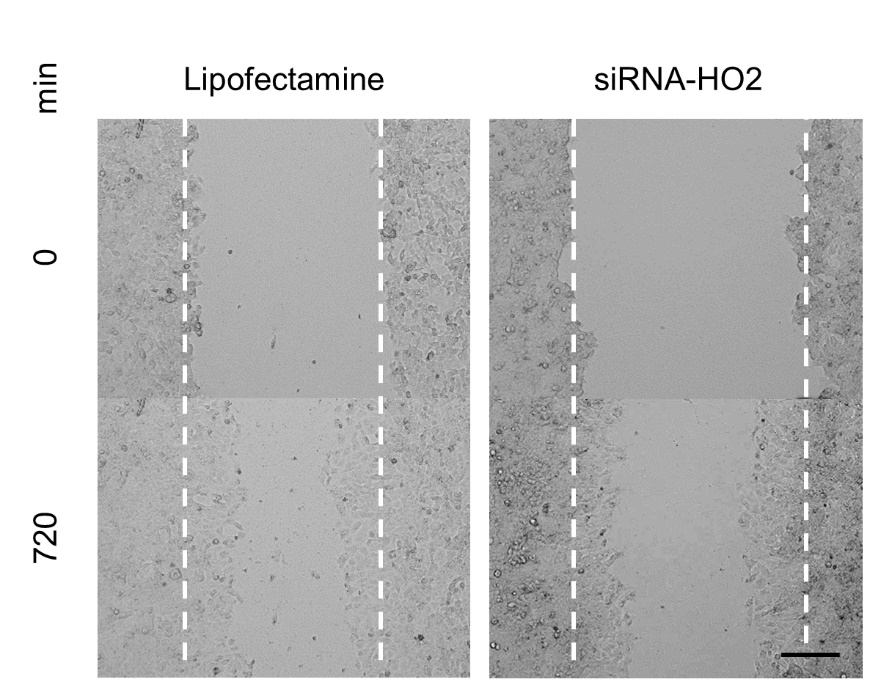


**Figures S2. Inhibition of A549 cell migration by regulating HO2 expression.** The migration of A549 cells on treatment with siRNA-HO2 was imaged using the EVOS M7000. Scale bars: 200 μm.


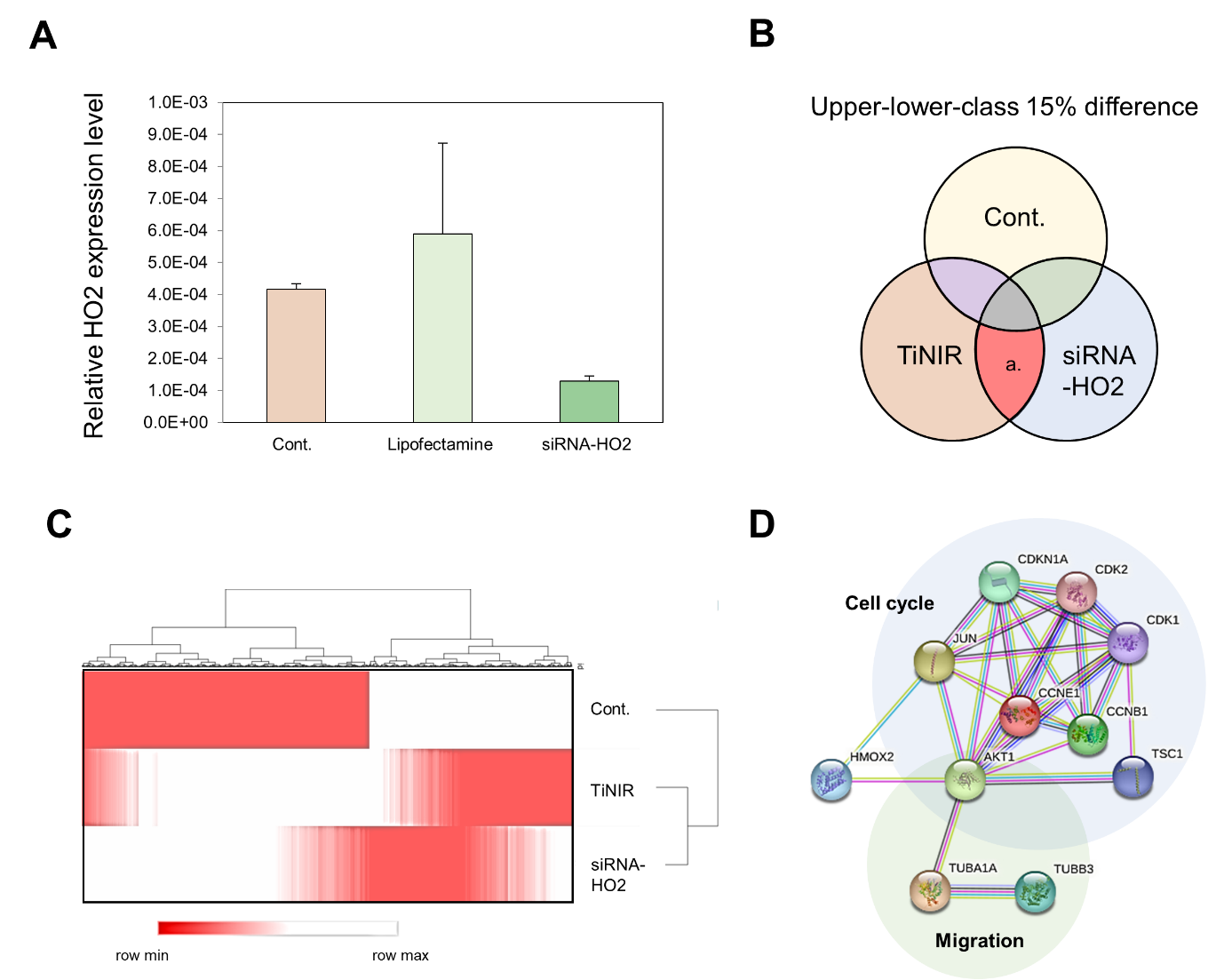


**Figures S3. Identifying expression of gene and interaction of co-related protein by inhibiting HO2. A** Confirmation of HO2 knockdown by siRNA-HO2. mRNA expression was measured by qPCR after treatment of siRNA-HO2 for 48 h (n=2, mean ± SD). **B** A total of 6,605 genes were extracted from the RNA-Seq list, selected by 15% upregulated and 15% downregulated mRNA (a) in the TiNIR-treated or siRNA-HO2-treated A549 compared to control cells. **C** Hierarchical clustering heatmap of the 6,605 genes. **D** AKT and cJUN, the linker of HO2 and related 8 proteins. The interrelation of AKT and cJUN with 8 proteins was calculated using PPI.


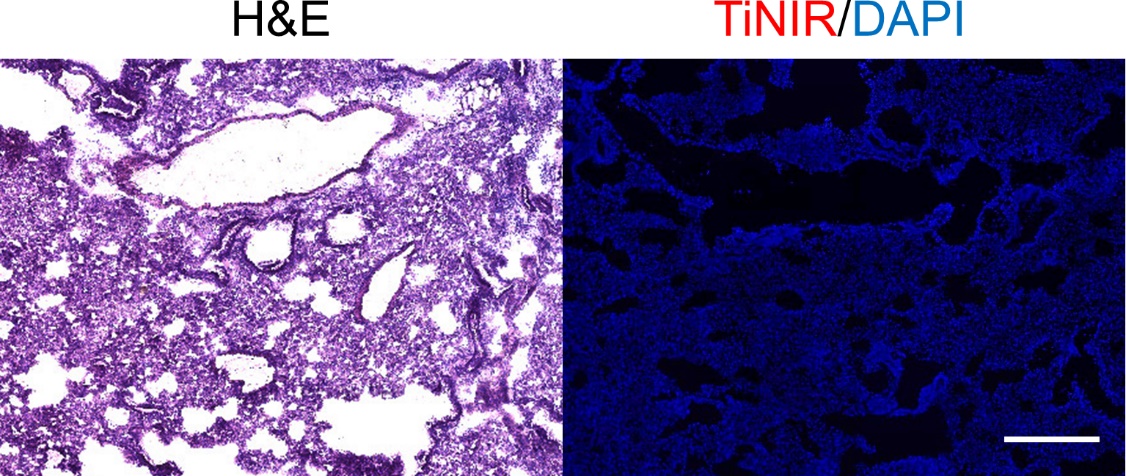


**Figures S4. The TiNIR signal in the normal mouse lung tissues.** The lung tissue was stained by H&E (left). Injected TiNIR signal in the normal lung tissue was investigated by fluorescent microscopy (EVOS M7000, ×40 air objective). Scale bars: 400 μm.

**Supplementary Video**

**Video S1.** Migration of A549 cells. The migration of A549 cells was recorded for 48 h under cell culture conditions (100% humidity, 5% CO2, 37°C, EVOS M7000, ×20 air objective). Scale bars: 200 μm.

**Video S2.** Inhibitory effect of the HO2 inhibitor TiNIR on A549 cell migration. The migration of A549 cells was recorded for 48 h after treatment with TiNIR under cell culture conditions (100% humidity, 5% CO2, 37°C, EVOS M7000, ×20 air objective). Scale bars: 200 μm.

**Video S3.** Single-cell migration of A549 cells. 3-D ODT time-lapse images of A549 cells were recorded for 24 h using the HT-X1. Scale bars: 14 μm.

**Video S4.** Inhibitory effect of TiNIR on single cell migration of A549 cells. A549 cells were treated with TiNIR, and a 3D ODT time-lapse image was recorded for 24 h using HT-X1. Scale bars: 14 μm.
